# Feasibility and effect of adding a concurrent parental component to a school-based wellness program using two modes of mobile-based technology – mixed methods evaluation of RCT

**DOI:** 10.1186/s12889-022-12581-7

**Published:** 2022-02-14

**Authors:** Moria Golan, Shakked Benifla, Aviv Samo, Noa Alon, Maya Mozeikov

**Affiliations:** 1 Department of Nutritional Sciences, Tel Hai Academic College, Kiryat Shmona, Israel; 2 Shahaf, Community-based body image and eating disorders facility, Ganey Hadar, Israel

**Keywords:** School-based, Prevention, Self-esteem, Body esteem, Mobile application

## Abstract

**Background:**

This study assessed the feasibility and effect of two mobile modes (WhatsApp vs. a specially designed app) in their delivery of updates and assignments to parents.

**Methods:**

Two three-armed, randomized, controlled feasibility studies were conducted. In each trial, four schools with a total of 418 students in grade 5th, mean age 10.1 years, were randomly allocated to the control arm, youth-only arm, or youth & parental component arm. Only the data of those that completed all three assessments (pre, post and 3 months post program) were analyzed: 133 in the first trial and 137 in the second.

In the youth-only arm, students participated in an interactive age-tailored prevention program delivered in 10 weekly, 90-min sessions on self-care behaviors, media literacy, self-esteem, and positive body image. The control groups in both studies received three health- and nutrition-related sessions.

In the parental arm, in addition to the ‘Favoring-Myself–Young’s ten sessions program, parents received updates and were requested to complete shared assignments with their children. In the first year, the assignments were sent via WhatsApp, and in the following year via “Favoring Myself” smartphone application.

Facilitators were third year undergraduate students. They used a detailed semi-structured guide and received 4-weekly hours of didactic and group dynamic supervision. Mixed-methods assessments were performed using semi-structured interviews with ten parents and five school staff members each year, as well as a computerized self-report questionnaire.

**Results:**

Feasibility of parent-adolescent shared assignments in both digital modes was lower than expected. The use of WhatsApp had higher feasibility and uptake than the use of the special application.

The addition of the concurrent parental component via WhatsApp was associated with superior improvement in self-esteem and identification of advertisement strategies, compared with the youth-only program. However, adolescents in the youth-only program delivered via the smartphone application demonstrated superior improvement compared to those in the youth and parental component arm.

**Conclusions:**

Although the addition of the concurrent parenting component was praised by the actively participating parents, overall, under the chosen structure and population, it did not prove to add statistically significant value to the youth-only arm.

**Trial registrations:**

NCT03216018 (12.7.2017) and NCT03540277 (26.4.2018).

## Background

The school years are a period of the life-course where young people are progressively at increased risk for mental health problems and educational disengagement [1, 2]. Prior research demonstrates the positive impact of parental involvement on children who participated in various school-based prevention programs [1, 3]. Moreover, relationship between parental involvement in youth programs and improved parent/child communication, bonding, and perceptions of one another was suggested. In addition, having common ground experience prolonged the positive post-participation effects of the intervention [4]. This evidence is consistent with Bronfenbrenner’s Ecological Systems theory, which views child development as a complex system of relationships, affected by multiple levels of surrounding environment; From the immediate setting of the family and school, to broader socioeconomic and cultural factors [5, 6]. It also implies that interactions with parents around learned topics at school might help address internalizing concerns [4, 7].

Although parents acknowledge the importance of children’s physical and mental health as well as changing attitudes toward increased parent-school collaboration, few parental component implementation studies on adolescents’ prevention programs in general and positive body and self-esteem in particular, have been conducted. A systemic review of the parental component in prevention programs to promote well-being reported that most programs included a minimal or unassessed parental component [8]. However, many of those that provided a more substantive intervention component for parents failed to recruit or retain sample sizes sufficient to allow statistical significance testing [8–10]. In programs that parents were highly engaged, the addition of a parental component was shown to improve students’ outcome [11]. When it comes to alcohol prevention, brief interventions focusing on both the parents and adolescents have shown better effects than those focusing only on the child or adolescent [12]. It seems that regarding addictive behaviors and interventions in which parents are approached with a brief parental component, as is common in the addictive field, participation may reach up to 80% - as was found in two out of the thirteen prevention programs with parental component [12].

While involving parents appears productive in theory, it is quite different in practice. Parents’ own negative school experiences, as well as low socio-economic status and/or low technology literacy, are likely to cause an undesirable impact due to limited uptake and lesser readiness to cooperate with school. Thus, they are likely to contribute rather than mitigate health disparities [13–15]. Further, engagement and the retention of parents in prevention programs are ongoing challenges. Parental attendance rates in family-based programs typically range from 35 to 50%, and up to one-third of those who enroll do not attend any of the sessions [15]. Only higher levels of parent-reported child mental health symptoms were associated with greater parental enrolment, and none of the fifteen assessed factors was found as a reliable predictor of parents’ ongoing engagement [15].

To address the notable disparities in access to evidence-based, cost-effective parenting interventions [16], and to overcome parental engagement barriers as well as enhance prevention program effectiveness, technology-based methods are employed in parenting programs [17]. The prominent use of smartphones among adolescents and their parents has led to an increase in health-related apps [18]. Moreover, digital interventions may also enable researchers to access geographically distant and busy parents [19], while addressing participants’ anonymity concerns. Although the efficacy of smartphone-delivered interventions is emerging, high rates of attrition and low adherence were reported, both of which threaten the validity of randomized controlled trial findings [20].

In school-based programs, the headteacher and senior management team encourage parental involvement, but parent-teacher differences in values, beliefs and expectations about what should be done and what is helpful, and lack of mutual trust and understanding were cited as barriers to successful family-school partnership [21]. Moreover, parenting involvement is successful when it is part of the school’s ethos and is developed and delivered as “a whole school approach” [21, 22]. Proactive relationship building with parents via telephone, or in-person pre-program contact was recommended to increase parental engagement. Further, providing update letters with shared assignments and adjunct support were some of the multi-component approaches suggested (e.g., information sheet, short video) [23].

As there is a lack of research on the best practices for parental engagement in shared assignments with their young-adolescents, the current feasibility studies assessed the effectiveness of implementing an evidence-based, school-based wellness program, with and without parental collaboration, through updates and shared tasks on adolescents’ self-esteem and body esteem.

The study primarily aimed to assess the feasibility and acceptability of two mobile modes in terms of delivering updates and assignments to parents: WhatsApp vs. “Favoring Myself- Young,” a specially designed application. We hypothesized that using the program’s specially designed application to contact parents and deliver the shared assignments would produce better uptake and parental engagement than when delivered via WhatsApp. The second aim was to assess the impact of the additional parental component on adolescents’ perceptions and behaviors. The main hypothesis was that adding a parental element to extend adolescents’ exposure and engagement to the prevention program’s topics would result in superior self-esteem and body esteem, self-care, and media literacy.

## Methods

### Design and sample recruitment

The two-year randomized clinical trial tested the feasibility, acceptability, and impact of “Favoring Myself-Young,” a manualized universal, interactive intervention program for young adolescents (5th graders) and their parents. Principals and school counselors of all fifteen elementary schools in northern Israel were contacted.

The eligibility criteria for schools included: a) having at least two 5th grade classes; b) agreement to schedule the program lessons on Mondays and Thursdays, due to the availability of the students facilitating the research; c) commitment to provide an appropriate room for each of the groups; and d) commitment to the presence of a class teacher or school counselor during the sessions to ensure appropriate behavior. In both years, four schools (out of the 15 contacted) were eligible and actually participated in the study.

### Randomization and blinding

Following the randomization protocol, eligible schools were randomly allocated by a research assistant blinded to their characteristics. Using the randomization function in Microsoft Excel, each year, schools were randomly assigned to one of the three study conditions: a) control condition, b) youth-only intervention, and c) youth with concurrent parental component condition. Schools were used as selection units to avoid contamination bias due to communication about the intervention between participants and controls within each school. Schools were blinded to condition allocation. Different schools were used for each year of the study. Differences in the baseline sample size in each arm were due to varying class sizes in each school.

### Study population

**Fig. 1:**
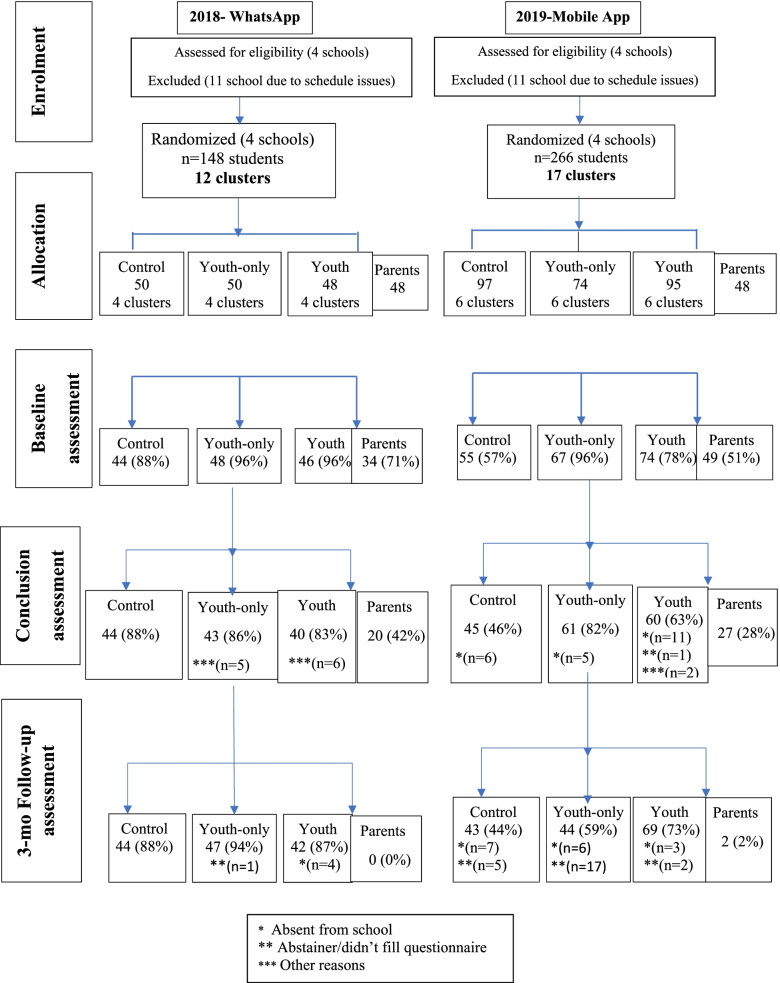
Flow chart of research participation in both studies

In the WhatsApp mode (first trial), 152 adolescents in the 5th grade were recruited for the program and 148 (97%) of them provided active consent of parents and students. However, 133 adolescents (89% of consenting participants) completed the three assessment questionnaires and were included in the final analysis. The participants’ flow is shown in the consort diagram (Fig. 1).

In the second study (with the specially designed application), 266 adolescents in the 5th grade were recruited for the program, 212 (80%) provided active consent of parents and students, and 137 (65% of consenting participants) completed the three assessment questionnaires and were included in the final analysis. No differences were found in personal characteristics between those that completed all three questionnaires and those that did not. Each class was divided into two equal groups for program delivery, comprising an average of 15 participants per group. There were eight intervention groups and four control groups in the first year (6 classes divided into two groups each), and twelve intervention groups and six control groups in the second year (9 classes divided into two groups each). Research students from the project team met with parents and staff of each class to recruit schools at parent-teacher conferences. Schools were provided with study information and letters of active consent. The participants’ flowchart is shown in the consort diagram (Fig. 1).

### Ethical procedures

The Tel Hai Academic College Institutional Review Board approved the research protocol (No 12/2017/− 1 and 08/2018–4). The trial methods and analysis strategy were pre-registered. The universal trial registration numbers are NCT03216018 (12.7.2017) and NCT03540277 (26.4.2018). Parents of students at all eligible schools received information about the program and the research study and provided informed active consent. All methods were performed in accordance with the Declaration of Helsinki and Consort 2010 guidelines and regulations.

### Interventions

#### Youth control arm

The control groups in both studies received three health- and nutrition-related sessions conducted by graduate students of Nutritional Sciences. These are regular information lessons, though not usually included in the school curriculum.

#### Youth intervention arms

“Favoring Myself-Young” is an interactive program comprising 10 weekly, 90-min sessions on self-care behaviors, media literacy, self-esteem, and positive body image. It is an evidence-based intervention that was empirically supported and substantiated with research findings that demonstrate beneficial and predictable outcomes [24] (Table 1). The program included a kit with background material, a detailed guide for facilitators with structured session plans, a framework for each topic, and interactive activities to engage participants verbally and non-verbally. To trigger situational interest, we used hands-on activities, novelty, surprise, and group work. Age-tailored games were often incorporated into the sessions. The program was semi-structured, with flexibility that enabled facilitators to be creative while addressing their groups’ specific needs, as was suggested by Slaten and Elison’s [25]. Group facilitators were undergraduate students of Nutritional Sciences and Education, who received two weekly hours of didactic training provided by the program’s founder, two hr group dynamic supervision and once a week personal supervision provided by an expert social worker throughout the intervention.

**Table 1:**
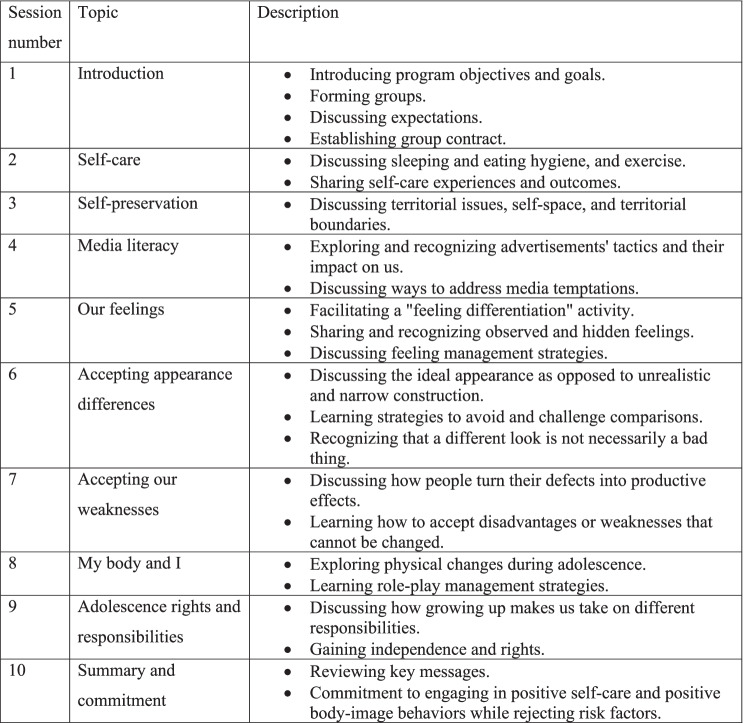
Content and description of the program sessions

#### Youth intervention with parental component

The parental component included weekly updates for parents about the current topic of discussion in the class. Moreover, one or two of the suggested shared assignments for parents and children were delivered concurrently with the “Favoring Myself-Young “class-based sessions. For instance, parents and children were required to describe two rights and two responsibilities the child gained in the last two years for the session on “growing up.”

Two reminders were sent to users who did not complete the weekly assignment. To motivate parents to engage and complete tasks, families received one “star” for each submitted assignment. Families who completed all tasks received a voucher for a family bowling game, to further encourage program adherence.

**Fig. 2:**
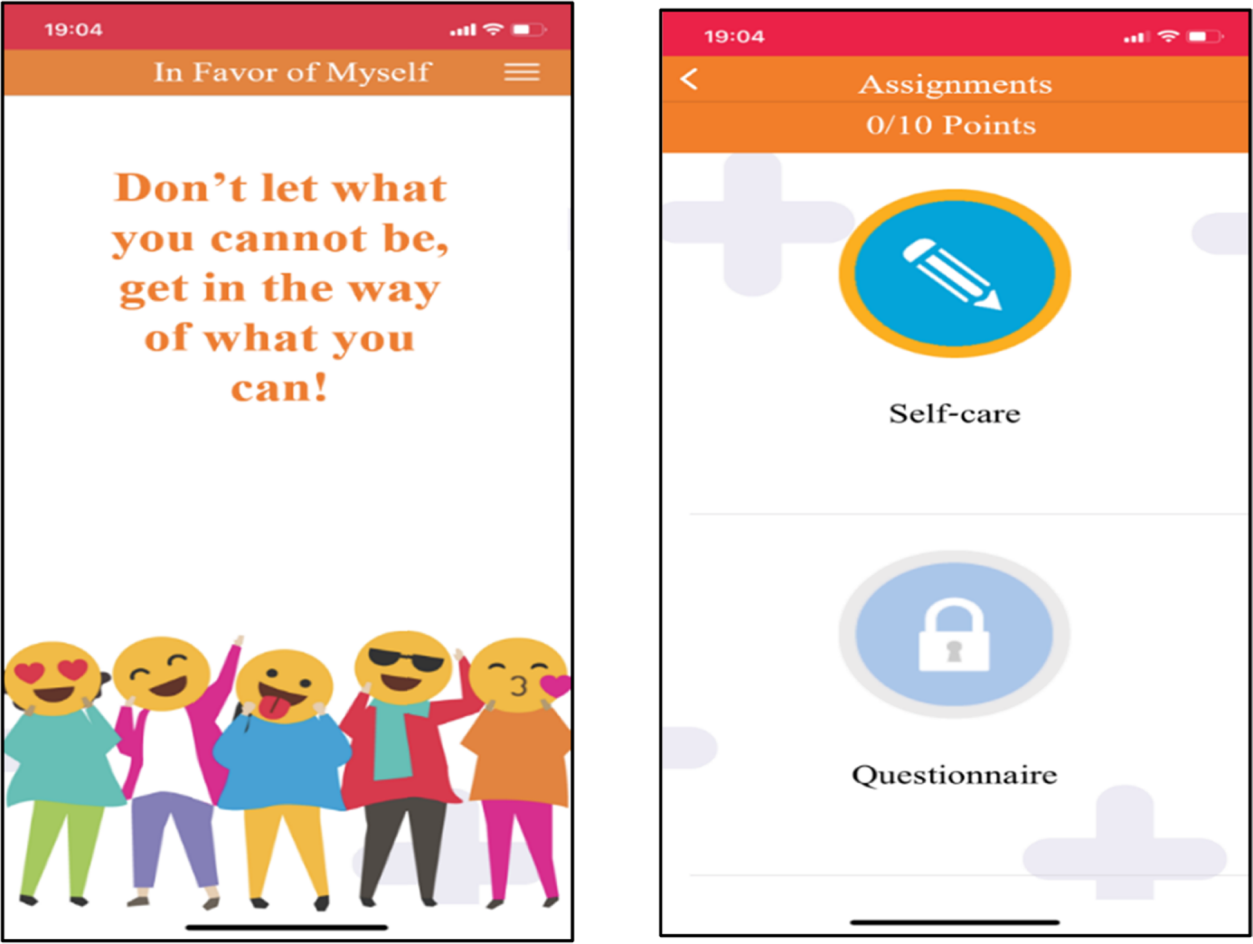
Screenshots (translated to English) from the “Favoring Myself” smartphone application

In the first year, the parental component was delivered via the WhatsApp application, while in the second year, it was delivered via the “Favoring Myself-Young” smartphone application, specially designed and programmed for this purpose (Fig. 2).

### Study questionnaire and data collection procedures

The computerized study questionnaire was completed online by participating students and parents via the Qualtrics XM Platform©, 2019. Students filled the questionnaires during school times in a computer classroom, under the supervision of program facilitators and a research student. Parents completed them via electronic link. Self-report questionnaires were preferred for participant confidentiality. Adolescents and parents completed the same questionnaire at three measure points – at baseline, program conclusion (two months later), and follow-up (three months after program conclusion).

Qualitative research aimed to better understand participants’ motives, incentives, and ideas about improving the program content, activities, or dissemination [26]. Personal and group semi-structured interviews were conducted with five selected schoolteachers and ten parents from the youth and parent groups at program cessation each year, to gain insights into program content, acceptability, satisfaction, implementation, and the perception about the effects it had on students, parents, and schoolteachers in various areas. Feedback about program strengths and limitations was invited. Of the interviewed parents, four were highly engaged with the parental component of the intervention, three refused to consent their child’s participation in the research, and three withdrew their consent for their child’s participation and their involvement in the parental component. The research team predetermined the semi-structured interview questions. Interviews lasted for 40 to 60 min and were conducted in interviewees’ home. All semi-structured interviews were audio-recorded and transcribed before the thematic analysis.

### Outcome measures and variables

Standardized instruments were used to measure program efficacy (Table 2). The pre-intervention questionnaire included demographic items on gender, age, familial status, and sociodemographic status. The post-intervention questionnaire consisted of a satisfaction assessment and an attendance report. All scales included in the study questionnaire were previously validated, Hebrew-translated versions.

**Table 2:**
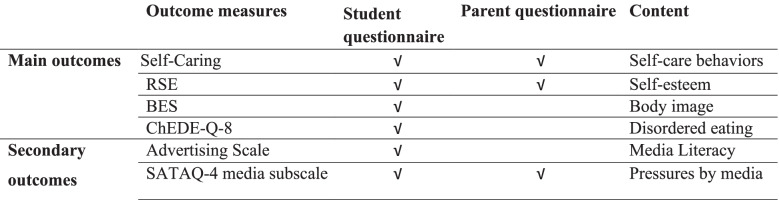
Overview of measures used to evaluate program efficacy in students and parents

*RSE* The Rosenberg Self Esteem Scale, *BES* The Body Esteem Scale, *ChEDE-Q-8* The Eating Disorders Examination Questionnaire-8 adapted for children, *SATAQ-4* The Sociocultural Attitudes Towards Appearance Questionnaire-4

### Outcome measures

**The Rosenberg Self Esteem Scale** [27]. The scale consists of 10 items rated on a four-point Likert scale. Items 1, 3, 4, 7, and 10 are scored from *strongly agree* (3) to *strongly disagree* (0), while items 2, 5, 6, 8, and 9 are reverse scored. The total scores range from 0 to 30. Scores from 1 to 25 indicate a normal range; scores below 15 suggest low self-esteem, while scores above 25 indicate high self-esteem. Cronbach’s alphas in these studies were 0.79, 0.74, and 0.81 for the control, youth-only, and parental component groups, respectively.

**Self-Caring Questionnaire**. Developed by Prof. Moria Golan (the first author), this questionnaire includes 20 items on personal and sleep hygiene, mealtime behaviors, and personal space. Items are rated on a five-point scale from *never* (1) to *always* (5) and higher scores indicate higher self-care behaviors [28]. The questionnaire was adapted for the parental version to include 20 additional items in which the parents responded according to their children’s as well as their own behaviors. Psychometric qualities assessed in a small pilot study (*N* = 10) revealed a Pearson Correlation Coefficient of 0.69 for reliability and Cronbach’s alpha of 0.85 for internal validity. However, in the current studies Cronbach’s alpha was only 0.66.

**The Body Esteem Scale** was used to assess body perceptions. The scale consists of 23 items divided into three subscales: appearance, weight, and attribution. The mean total and subscale scores are rated on a five-point Likert scale from *never* (1) to *always* (5). Higher scores represent higher body esteem [29]. Cronbach’s alphas in these studies were 0.79, 0.89, and 0.85 for the control, youth-only, and parental component groups, respectively.

**The Eating Disorders Examination Questionnaire**-8 adapted for children (ChEDE-Q-8) was used to assess eating disorder symptoms. The eight-item version of the original 28-item EDE-Q has excellent item characterization with high reliability. A strong correlation was found between the eight-item version and the original EDE-Q (*r* = 0.97, *P* < 0.001). For each statement, participants are asked to mark the frequency of occurrence in the past 28 days, and higher scores indicate a higher risk for eating disorders [30].

**The advertising scale** contains one item to measure participants’ identification of media strategies—a known protective factor. It includes eight different strategies, from which participants are required to choose; identifying a higher number of strategies indicates better media literacy [31].

**The ‘Pressures by Media’ subscale** of the Sociocultural Attitudes Towards Appearance Questionnaire-4 (SATAQ-4) was used to assess participants’ responses to media. Participants were instructed to rate their agreement with each item using a five-point Likert-type scale from *definitely disagree* (1) to *definitely agree* (5). A higher average score indicates higher pressures by the media to change one’s appearance [32].

All measures were used in both studies for parents and students. The Cronbach’s alpha reliability scores ranged between 0.67–0.89.

### Sample size and data analysis

The sample size was calculated for a moderate expected effect (*f* = 0.25), 80% statistical power, and an α level of 0.05, which was relative to the improvement in the Rosenberg Self Esteem Scale [33] and based on our previous data [24]. Calculation using G * Power software version 3.1.9.4 yielded a sample size requirement of 108 participants in all groups (parental component arm, youth-only intervention, control). Accounting for a 20% dropout rate, the total required sample was 120 participants in the three groups for each study. All analyses were conducted using IBM SPSS Statistics for Windows version 24, IBM Corp., Armonk, NY, 2017.

In the first study (WhatsApp), only those who completed the research questionnaire at two assessment times were included in the analysis. In the second trial (specially designed application), only those who completed the questionnaire at all three assessment times were included in the analysis, to prevent imputation bias. When data in this trial were analyzed with all participants who completed only two assessments, similar results were reached. The demographic variables between those who completed three questionnaires and those who completed less than three questionnaires were not statistically significant.

Data were checked for normality using histograms, scenes, and the Kolmogorov-Smirnov test through the Box test and Levene test. Sphericity was assessed through the Mauchly test and the assumption of the equality of variance-covariance matrices. The outcome data for the first study (WhatsApp) were analyzed using multivariate analysis of variance models by calculating the deltas between measure points (T2-T1, T3-T1) and using Bonferroni post hoc analyses between study groups.

In the specially designed application (second study), categorical demographic characteristics were presented as frequencies and percentages, and the association with study groups was tested using chi-square tests. Continuous demographic characteristics were presented as mean and standard deviation, and the differences between study groups were tested using the Kruskal-Wallis Test.

Most of the measures in this study (specially designed application) were not normally distributed and were therefore analyzed using non-parametric tests. Friedman tests were used to examine the differences between study times within each group, and Kruskal-Wallis tests were used to examine differences between the three study groups at each time point. Personal self-care hygiene and SATAQ-4 were tested using mixed model analysis. The effect size was marginal R^2^ for the mixed models, eta-squared for the Kruskal-Wallis Test, and Kendall’s W for the Friedman test.

For the qualitative analysis, all semi-structured interviews were audio-recorded and transcribed. The interview included questions on participants’ understanding of the program aims, barriers and facilitators to engagement, satisfaction from the topics and activities, and acknowledgments of change among their children and themselves.

The data were analyzed by identifying recurring key themes, which represented ways of understanding the combined meanings within the texts [34]. We chose a meta-ethnography analysis, widely used to qualitatively synthesize data in health and social care research [35]. Following the thematic analysis, we attempted to build a general interpretation based on the data This interpretive layer enabled deeper insights into the barriers faced in disseminating and implementing parental components in school-based prevention programs.

## Results

### Baseline descriptive characteristics of participants

In both studies, the mean age of all youth participants, both male and female, was 10.1 years (SD = 0.3). Groups appeared to be well balanced regarding baseline demographic characteristics (Table 3), as well as baseline outcomes values (Table 4). This was observed when data analyses were applied on the sample that included participants who completed at least two questionnaires as well as when only those who completed all three assessment questionnaires were included.

**Table 3:**
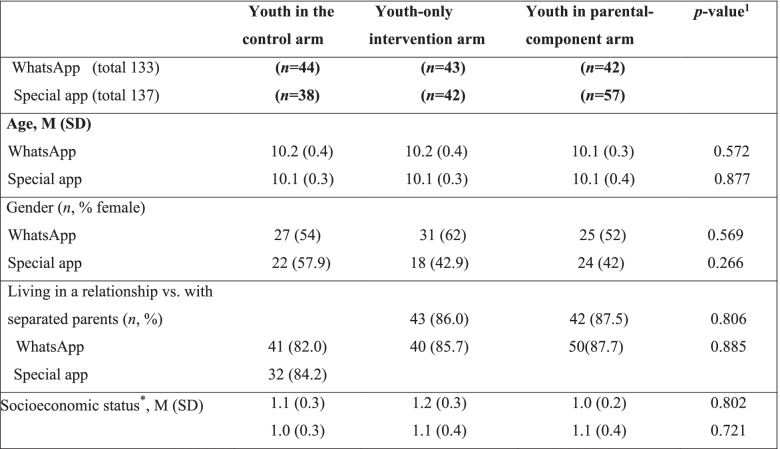
Baseline demographic characteristics of the study population in both digital delivery modes

*Calculated by the number of people per room in residence

M, mean; SD, standard deviation

^1^chi-square test for gender/parental status, Kruskal-Wallis Test for age/Socioeconomic status

**Table 4:**
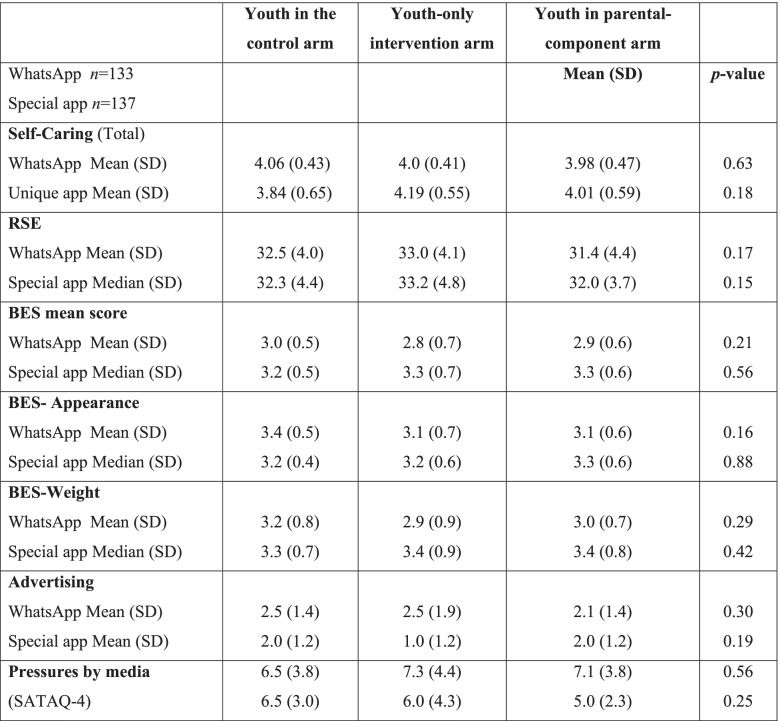
Adolescents’ baseline outcome measures in both digital modes

*M* mean, *Med* median, *SD* standard deviation, *RSE* The Rosenberg Self Esteem Scale, *BES* The Body Esteem Scale, *Eat-26* The Eating Attitudes Test, *SATAQ-4* The Sociocultural Attitudes Towards Appearance Questionnaire-4

### Responsiveness of adolescents and parents

**Fig. 3:**
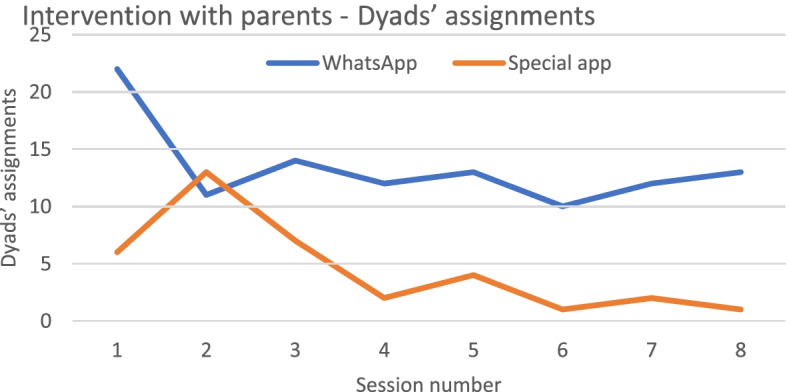
Number of Dyad’s shared assignments’ submission (parental component arm) delivered through WhatsApp vs. the special application

Results found that the responsiveness to program assignments deteriorated over time (Table 5 and Fig. 3). The concurrent parental arm demonstrated higher resistance to participate in the study, with the lowest percentage of consenting parents and the lowest rate of adolescents who completed the questionnaire at all assessment times, found in both digital modes.

**Table 5:**
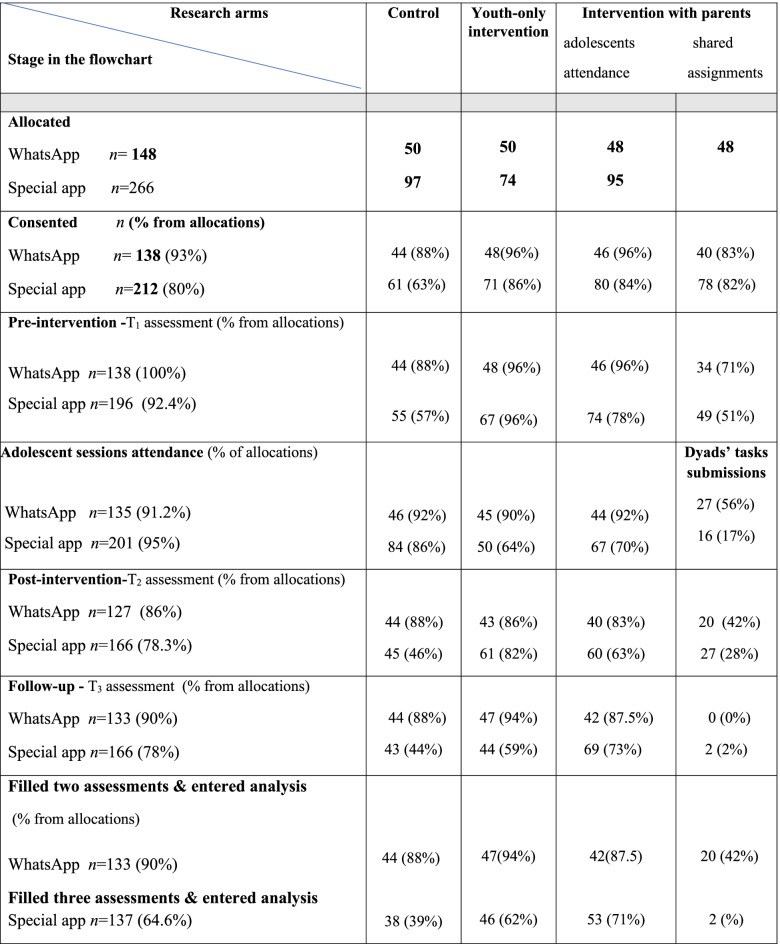
From allocation to analysis - number and percentages of participants in both digital modes along research stages

The WhatsApp mode of delivery was associated with 12% of allocated participants declining to provide active consent for their children to participate in the accompanying research. In comparison, 38% declined to consent when the specially designed application was employed.

Over time, the decrease in adolescent engagement was the lowest in the youth-only arm compared with the control group. A greater reduction in session attendance was found among adolescents in the parental component arm. Overall, the study groups and research stages show a significant superiority in participant responsiveness to WhatsApp as compared to the specially designed application (Table 5 and Fig. 3). No significant differences were found in baseline demographics and outcome characteristics between participants that completed the three research questionnaires and those that did not.

### Qualitative interviews

Conducted after program termination, these interviews shed light on parental and school team perceptions regarding addition of the parental component to the school-based wellness program. Five major themes emerged in the qualitative component of this study:

1. Most parents perceived school activity as a burden. “…leave us in harmony with our children, don’t interfere with the house schedule and type of conversation. I know you have good intentions, but I prefer if the school works within its territory and does not enroll me for shared tasks” (H.I.). “It is so hard to manage with four kids... the competing demands are my priority and not the suggested shared tasks…Moreover, my child is healthy; we don’t need it. Deliver it to parents with problematic children” (R.B.).
2. Rule enforcement. School teachers perceived the engagement of parents as necessary but preferred not to confront parents who expressed silent resistance. “The contents and the activities are great and age appropriate. However, most parents choose not to cooperate, and I prefer to choose my battles. If a parent chose not to cooperate, I will not penetrate his territory. I have to respect his avoidance. Personally, I admit, I am not particularly eager to cooperate when my child’s school enrolls me in activities” (M.C.). “I did not choose this program, and it does not address my priorities in being involved in the schools’ activities.” (B.R.)
3. Parental resistance to consent to participate in the research. Parents explained their resistance to participate due to privacy threat, fear of exposure, and law literacy regarding their and their children’s benefit from the research process. “Why should my child and I be like an experimental animal? If the program still needs research, I am not sure it is safe…” (A.R.). “I have heard from parents in the previous year that the research questionnaire is invasive. I will be honest with you; I don’t want you to wake sleeping dogs. Questions about how she feels about her body should be asked in a protective environment and not in primary school or on a research platform” (B.Y.).
4. Preference of WhatsApp usage over unique applications due to technological barriers and privacy issues. “At the beginning, I was glad the assignments were delivered via a digital platform, but when I failed to download the application, I decided that if someone wants me to participate. I prefer if it is sent through mail” (L.A). “I am overwhelmed with all the applications on my mobile; I don’t want another one that increases the chance to hack my phone – too dangerous in our times.”
5. High satisfaction was reported by parents and preadolescents who adhered to the shared assignments. “The topics in the program are vital for our children. The shared tasks were an excellent opportunity to talk with my child about how I handled age-related changes and how I react to stress” (B.C.). “These topics are so important, and to tell you the truth, in the classroom my child is too shy, and the shared assignment opened the door for him for intimate sharing and for me to get closer to him. Well done” (A.R).

### Intervention efficacy

#### Self-care

**Personal self-care hygiene** was not affected by the trial condition in either digital mode. A statistically significant interaction of group X time was found (*p* < 0.001; R^2^ = 0.03) with a small effect size with superiority in the control group (Table 6).

**Table 6:**
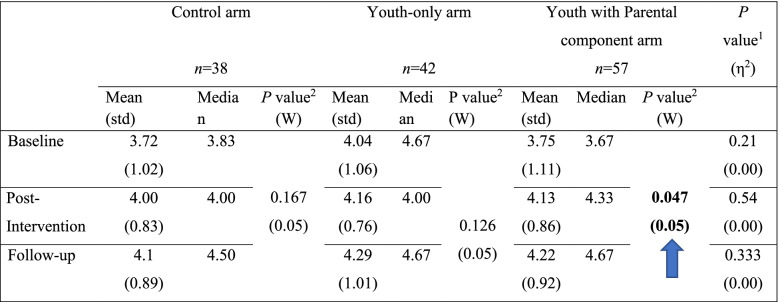
Special application mode: Self-care territory in the three groups over time

^1^Group effect (Kruskal-Wallis Test) – marked in bold the significant differences (after *p*-value correction).

Effect size: eta-squared interpretation is: < 0.06 (small effect), 0.06 - < 0.14 (moderate effect) and > = 0.14 (large effect).

^2^Time effect (Friedman’s Chi-Square Test) – marked in bold the significant differences (after p. value correction).

Effect size: Kendall’s W interpretation is: < 0.3 (small effect), 0.3 - < 0.5 (moderate effect) and > = 0.5 (large effect).

M, Mean; Med, Median.

**Self-territory care** was not affected by group or by times in either study. Nevertheless, in the specially designed application (second study), there was a statistically significant group X time effect with superiority (small effect size) in the concurrent parental component arm, along with all assessment times compared with the other two groups (Table 6).

### Self-esteem

**Fig. 4:**
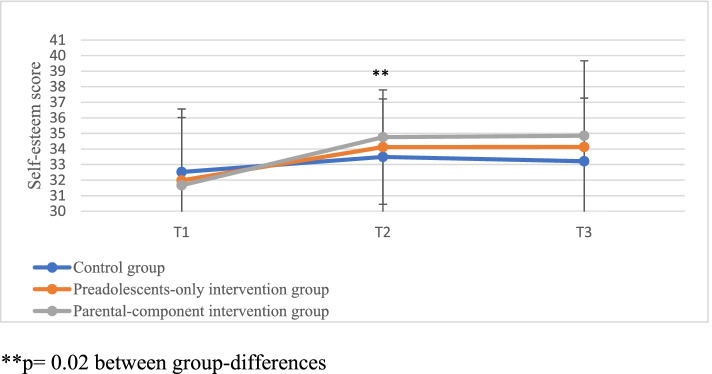
WhatsApp mode: Youths’ self-esteem in the three groups over time

In the WhatsApp mode (first trial), there was a statistically significant group effect with a small effect size (*p* < 0.005; η^2^ = 0.04) on youths’ self-esteem in the parental component arm at post-intervention. This improvement diminished at the three-month follow-up (Fig. 4).

In the second trial (specially designed application), a statistically significant change in youths’ self-esteem was also found at the follow-up assessment. The improvement was larger in the control (moderate effect size) and youth-only arms (small effect size), with no statistically significant change in the parental component arm (Table 7).

**Table 7:**
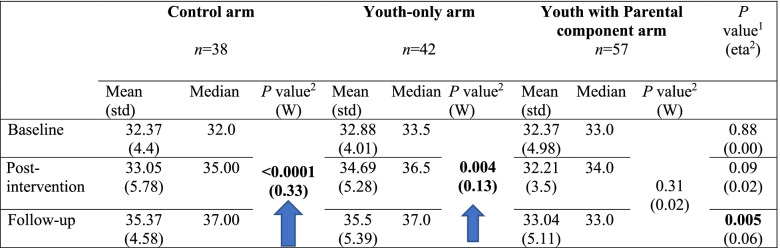
Special application mode: Youths’ self-esteem in the three groups over time

^1^Group effect (Kruskal-Wallis Test) – marked in bold the significant differences (after p-value correction).

Effect size: eta-squared interpretation is: < 0.06 (small effect), 0.06 - < 0.14 (moderate effect) and > = 0.14 (large effect).

^2^Time effect (Friedman’s Chi-Square Test) – marked in bold the significant differences (after p. value correction).

Effect size: Kendall’s W interpretation is: < 0.3 (small effect), 0.3 - < 0.5 (moderate effect) and > = 0.5 (large effect) M, Mean; Med, Median.

#### Body esteem

In the WhatsApp mode (first trial), there was no effect of trial conditions on either of the body-esteem subscales. In the specially designed application mode (second trial), there was a statistically significant effect of group on the body-esteem-appearance subscale, at program termination, with statistically significant superiority in the youth-only arm at post-intervention as well as at the follow-up assessment (small effect sizes). In the weight subscale, there was no group or time effect. However, there was a statistically significant interaction of group X time with superiority in the control group at the follow-up assessment, with moderate effect size (Table 8).

**Table 8:**
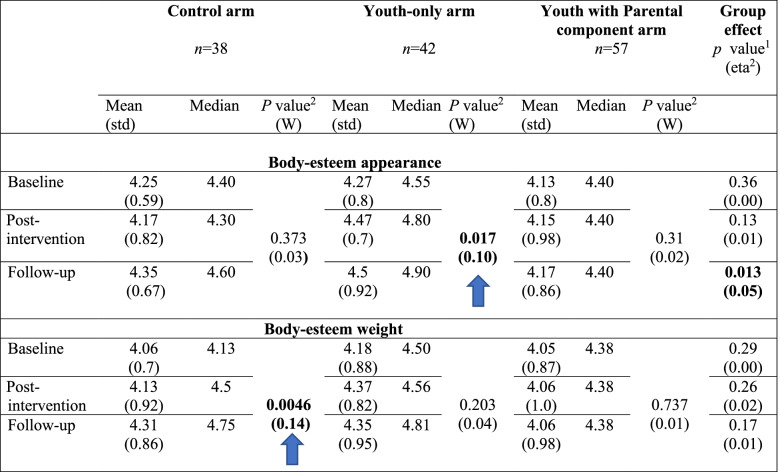
Special application mode: Youths’ body-esteem in the three groups over time

^1^Group effect (Kruskal-Wallis Test) – marked in bold the significant differences (after p-value correction).

Effect size: eta-squared interpretation is: < 0.06 (small effect), 0.06 - < 0.14 (moderate effect) and > = 0.14 (large effect).

^2^Time effect (Friedman’s Chi-Square Test) – marked in bold the significant differences (after *p* value correction).

Effect size: Kendall’s W interpretation is: < 0.3 (small effect), 0.3 - < 0.5 (moderate effect) and > = 0.5 (large effect).

M, Mean; Med, Median.

#### Media literacy

Media literacy was assessed using the number of advertisement strategies identified and the perception of being influenced by media pressures to change one’s appearance.

**Fig. 5:**
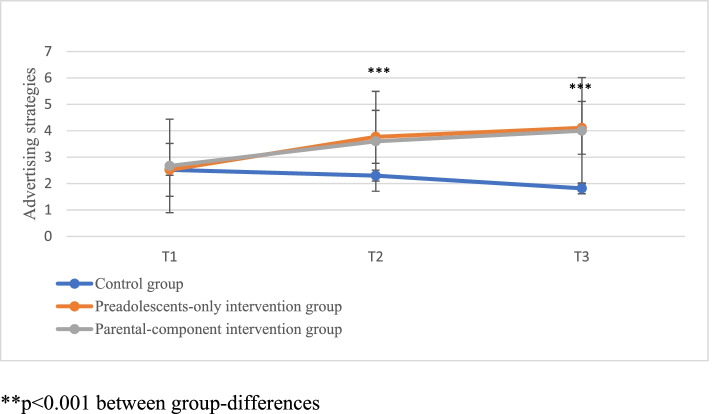
WhatsApp mode: Number of advertising strategies identified in each study arm over time

In the WhatsApp mode (first trial), the youth-only arm and the concurrent parental component arm demonstrated a statistically significant superiority and increase in identifying advertisement strategies over time compared with the control arm (Fig. 5) with a large effect size (η2 = 0.264). No effect of trial conditions was found on the perceived pressure experienced by the media to change appearance (SATAQ-4).

.

In the specially designed application mode (second trial), there was no effect of trial conditions on the number of advertisement tactics identified by youth and on perceived pressure imposed by the media to change appearance (SATAQ-4). Nevertheless, when youth responses were divided into those under 1 (not affected by media) and above 1 (affected to a large extent), the analysis revealed a decrease of 29% in the control group among youth being affected (from 73.7% at baseline to 44.7 at follow-up). Moreover, a 31% reduction (from 64 to 33%) in the youth-only arm and a 14% reduction in the parental component arm (from 68% at baseline to 54% at follow-up) were found. Mixed model analysis revealed a statistically significant effect of group (χ^2^ = 6.44; *p* < 0.05), time (χ^2^ = 20.96_**;**_ *p* < 0.001), and time X group (χ^2^ = 13.27_**;**_ *p* < 0.01).

#### Eating disorders behaviors

There was no effect of trial conditions or study arms on the eating disorders’ behavior scores. At all assessment points, the scores were far below the cut-off for identifying pathology in this age group.

## Discussion

There is robust evidence of the associated positive outcomes of parental engagement in school-based prevention programs [36–38]. Digital-based prevention programs were suggested for this purpose [11, 16, 39].

The current study examined the feasibility and impact of concurrent parental components (parents receive updates and shared assignments with adolescents) integrated into “Favoring Myself-Young” a school-based interactive wellness program for 5th grade primary school students. The parental component includes weekly topic update letters to parents, with suggestions for dyad-shared assignments. A prior study has suggested this strategy to avoid parents from falling into the role of disciplinarians [4].

The current study revealed that parents’ engagement in the shared assignments was short-term only and deteriorated during trial times. Participants’ attrition presents a significant problem for even the most well-planned and well-executed intervention projects with internal and external validity and facilitator motivation. For instance, Amaral et al. reported that 50% of intervention participants attended all four prevention program sessions and completed the posttest assessment [40]. Morgan et al. reported that only 38% of the parents that participated in mental health first aid training completed the 3-yrs follow-up assessment [41].

However, the retention rate was higher when the concurrent parental component was delivered by WhatsApp than by a specially designed application. The application was designed to create an intimate environment where parents could consult the specialist who developed the shared assignments, and ask personal questions related to the discussed topics, anonymously or openly. Moreover, this platform was intended to serve facilitators by sending memos and funny responses to generate continuity and engagement with the program topics. In contrast to our hypothesis and expectations, parents expressed resistance to downloading the specially designed application.

Although parents believed in the program’s importance and shared the school’s interest in preventing risk behaviors, they did not perceive the program as being their choice or as reflecting their needs and interests. Although most parents (not all) provided active consent, they easily withdrew their consent when it came to implementation.

Barriers, such as technical difficulties, fear of exposure, concerns about privacy, and lower motivation to collaborate on shared assignments were explored retrospectively through qualitative interviews. Similar barriers were reported in studies that implemented parental components in school-based prevention programs [42]. These barriers may be attributed to the rapid proliferation of social networking that has transformed the way people socialize and communicate [43]. Parents, like other human beings, are more concerned with their freedom and privacy owing to the developed vigilance toward technology and media penetrations. As was shared in the personal interviews, parents tend to develop negative attitudes to experimental research and prevention programs that were not personally chosen by them, sometimes unconsciously. Relying on the collaboration with schoolteachers was one of the program barriers. From the perspective of an external supplier, the partnership between parents and schools felt weak, and contributed to the barriers in implementation and adoption of prevention programs. The shift from worshiping the collective and the community to individuality in western societies [44] is also expressed in the imbalanced parent-school partnership. It seems, as years go by, the relationship between parents and the education system shapes the feasibility and effectiveness of school-based prevention programs. Often, shared parent-child school-based assignments tend to trigger responses to the intervention itself, thus failing to address the program objectives [45]. In the described project, schools’ management limited the research team’s access to parents due to their extreme caution of burdening parents. Under these circumstances, the idea of creating a public partnership to establish a better platform for the program fade away. In contrast to the current study hypotheses and others’ findings, our statistical comparisons revealed that the addition of the parental component was not statistically superior to the youth-only arm. Thus, under the chosen structure and population, the program did not have the intended impact. Moreover, parents’ resistance to the parental component may have induced negative attitudes among their children, thus diminishing their ability to be positively influenced by the program content, as seen in the described trials’ effects.

Morgan et al. (2020), who studied the long-term effect of a prevention program, reported that between baseline and 3-year follow-up, there was a non-significant reduction in adolescent cases of mental health problems relative to the control group (odds ratios (OR) 0.16–0.17), a non-significant improvement in parental support reported by adolescents with a mental health problem (OR 2.80–4.31), and a non-significant improvement in the quality of support that parents reported providing to their adolescents with a mental health problem (d = 0.38). The only maintained achievement included parents’ improved knowledge about mental health problems [41]. Both Morgan’s et al. and our results question the contribution of parental components in school-based programs.

Further, it seems that WhatsApp was preferred by adults over the specially designed application. This may be attributed to the perceptions that WhatsApp is more intimate [46]. Moreover, the specially designed application provoked resistance due to the need to download “one more temporarily used item to the mobile phone” as was suggested in the qualitative interviews.

Overall, the study groups and the research stages showed a significant superiority in participant responsiveness to WhatsApp over the specially designed application. The concurrent parental component via WhatsApp was associated with statistically significant improvements in only two measures out of the seven outcome variables, and only one statistically significant improvement in the youth-only arm. Nevertheless, when it was delivered via the specially designed application mode a statistically significant improvement was found only in one out of the seven measures.

The youth-only arm showed statistically significant improvements in three out of the seven outcome measures compared to four statistically significant improvements found in the control group, which received a shorter intervention but demonstrated higher attrition which may lead to higher false-positive outcomes in this group.

The small effect size of most changes in this study may suggest that a larger sample size, more intensive exposure and new ways to create community-based participatory research are needed for future assessments..

### Strengths and limitations

This study has several strengths. First, two active control groups were used; one youth-only group, without the parental component, and another with a shorter intervention to control expectancy effects. Second, the combination of quantitative and qualitative research methods deepens the understanding of the implementation of the parental component arm. Third, the repeated facilitation of the same prevention program and the continuity search for expansion of program exposure to students, is another strength.

The current study has a few limitations. First, as mentioned before, attrition bias may increase false-positive outcome. Despite this, no effects on outcomes were found in comparing the analyses between participants who submitted the shared assignments and those who did not, as well as in the analyses between those who completed two vs. three questionnaires. Second, the findings of this study are based on self-reports, which could be subject to social desirability bias. Additionally, our results may not be representative of the preadolescent population, as this study was performed with a selective population in a small country. Thus, conclusions should be interpreted with caution. Nevertheless, the consistent non-significant effect of the experimental parental component on most measures supports the robustness of the findings, despite the limitations and the fact that the study findings contrasted with the research hypothesis.

## Conclusions

The use of the WhatsApp application had higher feasibility and uptake than the use of the specially designed application. Although parents and school team members expressed a positive stance toward shared assignments, poor uptake, dropout, and noncompliance may hinder the validity of our findings. Moreover, the relatively low rate of parental cooperation and the small effect sizes raise questions regarding the cost-effectiveness of adding a parental component to universal school-based prevention programs. Future studies should strive to overcome parental resistance as well as improve parent-school collaboration and. Moreover, a cost-benefit analysis of parenting programs is critical for scaling up prevention programs.

## Data Availability

The data that support the findings of this study are available from the first author upon reasonable request and with permission of The College IRB. Restrictions apply to the availability of these data by the ministry of education, and so are not publicly available.

## Abbreviations

RCT: randomized clinical trial
app: application
ChEDE-Q-8: The Eating Disorders Examination Questionnaire-8 adapted for children SATAQ-4 Sociocultural Attitudes Towards Appearance Questionnaire-4
OR: odds ratios
d: d cohen effect size
